# Human Physiology, Statistical and Dynamical; or the Conditions and Course of the Life of Man

**Published:** 1856-10

**Authors:** 


ART. IV.—*Human Physiology, Statistical and Dynamical; or the Conditions and Course of the Life of Man.* By John Wm. Draper, M. D., LL.D., Professor of Chemistry and Physiology in the University of New York. Illustrated with nearly 300 Wood Engravings. New York: Harper & Brothers, Publishers. 1856.

Professor Draper publishes, in this book, the lectures on Physiology which he had delivered to his class for many years past, with such changes as are rendered necessary by the advance of the science, or the change in the manner of conveying the instruction.

It is needless to say that the volume is an able one. Dr. Draper has both talent and learning to make it such. And we can recognize in it the chemist, predominant over the physiologist. Of late years, the problems of vital chemistry have forced themselves within the pale of physiological discussion, and, of course, Dr. Draper, the chemist, does not yield in this regard to Dr. Draper, the physiologist. Like any other chemist, he has more faith in the solvent power of acids, or the destructive agencies of fermentation, than in such "*nonentities*" (we use his own word) as irritability, vital power, plastic force. He would cast these out from the nomenclature of physiology. Dr. Draper is a disciple of Positivism. "Physiology alone," he says, "of all the great departments of knowledge, retains the metaphysical conceptions of the Middle Ages, from which Astronomy and Chemistry have made themselves free." * * * * * * "Metaphysical Philosophy has lost its hold upon the human mind. The uncertainties, contradictions, and emptiness of the English, Scotch, French, and German schools are manifest."

It would undoubtedly be a step in advance to get rid of those ideas of mental philosophy, which stood as a substitute for physiology when Abercrombie taught, or when Unzer, a hundred years ago, first propounded the idea of reflex action, since developed by Marshall Hall, or that of "involuntary cerebration," now embodied in all theories of the functions of the brain. True it is, that some of the most valuable truths of science were first developed in these theories; but, so far as we can trace the agencies which gave them birth, they are the product of whatever experimental knowledge then existed, and not of logical induction.

To our notion, Dr. Draper is right in wishing to cast out from modern physiology everything but exact, proven facts. Such a subtraction from the science would be a serious dispensation to the idealists, who get up such windy talk about "objective" and "subjective," and muddle their brains over innate ideas until they grow into a tipsy, æsthetic state, in which they wander about like babes in the woods, in hopeless bewilderment.

Let physiology start fair. Garner her facts and let *them* be the science of physiology; until enough of them are gathered to frame a comprehensive theory of life, which shall harmonize them all, and show us that beautiful unity in diversity which is the true law.

We have not space in this crowded number to do justice to his book. His reputation as a teacher is such as to bear with it weighty recommendation —his adherence to proven fact, and his rejection of vague theory and hypothesis, constitute a still higher merit.

For sale by PHINNEY & Co.

